# Occurrence, multidrug resistance and potential risk factors for *Staphylococcus aureus* infection at worker-animal and working equipment interfaces: a systematic review and meta-analysis of the Ethiopian literature

**DOI:** 10.3389/fpubh.2024.1403012

**Published:** 2024-08-16

**Authors:** Bemrew Admassu Mengistu, Kalkidan Getnet, Atsede Solomon Mebratu, Melkie Dagnaw Fenta

**Affiliations:** ^1^Department of Biomedical Science, College of Veterinary Medicine and Animal Science, University of Gondar, Gondar, Ethiopia; ^2^Department of Veterinary Epidemiology and Public Health, College of Veterinary Medicine and Animal Science, University of Gondar, Gondar, Ethiopia; ^3^Department of Veterinary Pharmacy, College of Veterinary Medicine and Animal Science, University of Gondar, Gondar, Ethiopia; ^4^Department of Veterinary Clinical Medicine, College of Veterinary Medicine and Animal Science, University of Gondar, Gondar, Ethiopia

**Keywords:** animals, humans, multidrug-resistant, *Staphylococcus aureus*, working equipment

## Abstract

**Background:**

*Staphylococcus aureus* (*S. aureus*) infecting animals and humans via close contact, handling, or consuming contaminated products is a growing public health concern. In Ethiopia, it is important to examine the overall prevalence of *S. aureus*, patterns of multidrug resistance, and potential risks in human-animal interface settings. Thus, this review was conducted to estimate the pooled prevalence of *S. aureus*, its multidrug resistance, and potential risk factors for worker-animal-working equipment interactions.

**Methods:**

This systematic review and meta-analysis were carried out by the PRISMA guidelines. The research articles were searched from PubMed, HINARI, Web of Sciences, and Google Scholar databases.

**Results:**

This meta-analysis included 13 independent articles and 52 dependent studies. In total, 5,329 humans, 5,475 animals, and 5,119 samples of working equipment were analyzed. The pooled prevalence of *S. aureus* at the interfaces between humans, animals, and working equipment was 22%, there was a high level of heterogeneity (I^2^ = 94%: *p* < 0.01). The overall pooled prevalence of *S. aureus* in dairy farm sources was 23% (95% CI, 17–30%) compared to 18% in abattoirs. The pooled prevalence of *S. aureus* was estimated to be 25% for human sources, 23% for animal sources, and 19% for working equipment. The total multidrug resistance (MDR) rate was 27%. The present study illustrates that a predominant antimicrobials comprising ampicillin, penicillin, chloramphenicol, tetracycline, and ciprofloxacin, accounts for the development of resistance in *S. aureus* strains, with a prevalence of 72%. According to the qualitative assessment of potential risk factors, animal age, worker education, lactation stage, and hand washing by milkers influenced the circulation of *S. aureus* at animal-worker and working equipment interfaces.

**Conclusion:**

The pooled prevalence of *S. aureus* at the interface of human,-and animal-working equipment was quantified at 22%. *S. aureus* was found in humans, animals, and equipment at nearly the same rate. The results of this study demonstrate that *S. aureus* is hazardous and circulates among animals, workers, and equipment: farmers, animal owners, employees, and the public need to be educated about *S. aureus*. Moreover, animals and work equipment should be included in the control and prevention of *S. aureus* infection.

## Introduction

In pastoral environments characterized by close human-animal interactions, *Staphylococcus aureus* has emerged as a zoonotic concern of public health importance due to the prevalence of multidrug-resistant microorganisms. It has long been recognized as a significant concern in terms of public health (1). This bacterium, which exhibits a positive Gram stain, displays a vast geographic dispersion, as evidenced by investigations conducted in diverse regions across the globe (1, 2). The frequency of food products contaminated with *S. aureus* varies, with cereals having the highest frequency and meat and bean products having the lowest. The hospital and community settings have been the sites of methicillin-resistant *S. aureus* (MRSA) strains, with several dominant clones reported in various countries (3, 4). Antibiotic-resistant genotypes and host epidemiology have combined to cause the spread of community-associated MRSA lineages, changing the transmission dynamics and resulting in sustained transmission in regional population centers (1, 5).

In addition to causing zoonotic diseases, it can lead to mastitis, bacteremia, and food-borne poisoning in humans (1, 6). Staphylococcal enterotoxins are a prominent source of food-borne outbreaks that are frequently linked to *S. aureus* (5, 7). Since *S. aureus* frequently co-occurs with humans, care must be taken to lower the possibility of contamination when preparing food. The presence of biofilm-related medical equipment infections is also a significant issue. Currently, methicillin-resistant *S. aureus* infections have become endemic worldwide (1, 2, 7).

Research has indicated a significant frequency of *S. aureus* colonization among employees in various work environments, such as bakeries, pig farms, fire departments, and healthcare facilities (4). Investigations into the presence of *S. aureus* in cows, their handlers, and their immediate environment have also revealed a diversity of clonal lineages and suggested a potential route of transmission between handlers and cows (4, 8). As it has been pointed out in the different studies, there is a potential occupational contact with swine, such as that experienced by hog slaughterhouse workers, influences nasal *S. aureus* colonization (9, 10). Among the antibiotic resistance genes, variations in lineage distribution and virulence factors of *S. aureus* have been studied in pig workers compared to non-workers. Due to their close contact with livestock treated with antibiotics, pig workers are at higher risk of carrying antibiotic-resistant *S. aureus* (11). Methicillin-susceptible *S. aureus* (MSSA) strains with variable virulence factors and antibiotic-resistance genes have been detected in swine populations (11, 12). Furthermore, research on the epidemiology and genetic relatedness of the *mecA* gene in *S. aureus* strains isolated from pets, contact persons with pets, and veterinary clinical environments has revealed the increasing prevalence of methicillin-resistant *S. aureus* (MRSA) strains at animal-human-environment interfaces (5).

Staphylococcal Cassette Chromosome *mec* (SCC*mec*) is indeed a critical genetic element responsible for the multidrug resistance in *S. aureus* strains, particularly methicillin-resistant *S. aureus* (MRSA). SCC*mec* is a mobile genetic element that carries the *mecA* gene, which encodes penicillin-binding protein 2a (PBP2a). PBP2a has a low affinity for β-lactam antibiotics, including methicillin and other β-lactam antibiotics, rendering the bacteria resistant to these drugs (13). Methicillin-resistant *S. aureus* is connected to hospitals, and the population is most commonly associated with type IV *SCCmec* elements, which are categorized into 15 categories (14). The whole-genome sequence of the *SCCmec* IVd (2B) subtype, one of several subtypes of *SCCmec* type IV strains, is still missing (13). Additionally, *S. aureus* has multiple transmissible genes, including plasmids and transposons. These genes spread antibiotic resistance genes between *S. aureus* strains that confer resistance to various antibiotics through mechanisms like enzymatic inactivation, altered target sites, and reduced intracellular drug accumulation (15). Little is known about the processes that facilitate the acquisition of foreign genes and the dynamics of their transfer between hosts (16).

Workers may be susceptible to colonization by *S. aureus* in a variety of work contexts. There is a high incidence of *S. aureus* colonization among healthcare workers (HCWs) in several nations, including Indonesia (17), Portugal (18), Ecuador (19), and Nigeria (20). Healthcare professionals who have previously worked in a hospital with a positive MRSA colonization history are at risk for *S. aureus* colonization (21), and other risk factors include not practicing hand hygiene, being male, being older, not washing your hands well, having a wound or skin infection, and recently using antibiotics (22–24).

Because *S. aureus* has a high incidence of antimicrobial resistance, it presents substantial clinical danger in Africa. Methicillin-resistant *S. aureus* (MRSA) is a contributing factor to hospital-associated and community-associated infections in Africa. Africa has yielded several clonal complexes (CCs), demonstrating the diversity of the clonal spread of MRSA across the continent. The three most frequently used MRSA strains are the ST5-IV [2B], ST8-IV [2B], and ST88-IV [2B] clones (3). Many CCs have MRSA that is Panton-Valentine leukocidin (PVL)-positive meticillin-resistant *S. aureus* and the frequency of PVL-positive MRSA varies from 0 to 77% in Africa (25). Inducible clindamycin resistance in *S. aureus* isolates varies from 2.9 to 44% in Africa; MRSA strains have a greater incidence of this resistance (26). Regarding the significance of antibiotic resistance in *S. aureus*, routine screening, prudent clindamycin usage, and molecular identification of resistance genes are advised (24, 27, 28). Kenyan community-associated MRSA (CA-MRSA) strains have been characterized by whole-genome sequencing, which has led to the discovery of rare sequence types (STs), such as ST7460 and ST7635 (28). Different *spa* types and clonal complexes have been identified by combining DNA microarray and *spa* typing to characterize *S. aureus* isolates from South Africa and Nigeria (3, 25, 29, 30).

Studies show that *S. aureus* is prevalent in a variety of contexts and is a major concern in Ethiopia. The country has a 23% overall pooled apparent prevalence of *S. aureus* in milk and meat samples according to a comprehensive study and meta-analysis (31). Another study that examined lines of cattle abattoirs revealed that 35.5% of the isolates were *Staphylococcus*, and 13.6% of those isolates were *S. aureus* (32). *S. aureus* is the most often isolated bacterium in burn wound infections, with an overall incidence of 57.8% (33). Methicillin-resistant *S. aureus* (MRSA) and other various strains were identified by molecular analysis of *S. aureus* isolates, underscoring the possibility of pandemic strains in the nation (34). Additionally, samples of cottage cheese and yoghourt included *Staphylococcus* species, with an overall prevalence of 14.3% and a specific prevalence of 22 and 6.5% in the former and yoghourt, respectively (35). Numerous researchers have examined the status of bacteria and the risk factors that are connected to the interface between personnel, animals, and working equipment, with a focus on Ethiopia (36–38). These results highlight the need for better sanitation habits, antibiotic stewardship, and public education to lower the prevalence of drug-resistant *S. aureus* infections in Ethiopia.

Thus, to support successful preventative and control initiatives, it is imperative to understand the general occurrence, multidrug resistance rate, and potential risk factors for *S. aureus* at the workers-animal-working utensils interface at the national level. Accordingly, this meta-analysis aimed to gather the available data and estimate the pooled prevalence of *S. aureus* and its antimicrobial resistance at the workers-animal-working interface in Ethiopia.

## Methods

The PRISMA (Preferred Reporting Items for Systematic Reviews and Meta-analysis) checklist (39) was used to conduct this review.

### Search strategy

The literature search took place between October 2023 and November 28, 2023. A careful search technique was developed to conduct a comprehensive review of all relevant studies (B.A., and A.S). Various databases, including PubMed, Google Scholar, HINARI, Web of Science, and Snowball search engines, were used for retrieving articles, and other manual methods were also used to conduct the literature search to select the remaining included studies. This systematic review and meta-analysis used the CoCoPop (Condition, Context, and Population) and PEO (Population, Exposure, and Outcome) frameworks to search for relevant articles. The disease studied was *Staphylococcus aureus* (*S. aureus*) (Co), the context was Ethiopia (Co), and the population consisted of both animals and humans (Pop). The PubMed search strategy included Medical Subject Heading (MeSH) terms and a wide range of important keywords.

From an epidemiological perspective, *S. aureus* is a globally widespread, zoonotic, and transmissible bacterial infection that affects both humans and animals. The research question was formulated as follows: “What is the prevalence of *S. aureus* and associated risk factors at the interface between workers, animals, and equipment in Ethiopia?” The Boolean operator “AND/OR” was used for the online search, and we used this operator to identify relevant results by combining similar phrases and words. The search terms used were (*Staphylococcus aureus* OR *S. aureus*) AND (epidemiology OR prevalence OR infection rate) AND (cattle) AND people AND work equipment AND risk factors OR predisposing factors AND (Ethiopia). The language of the publications was limited to English. All identified studies were imported into End Note 20 software to avoid duplicate entries.

### Operational definition

#### Interface

A place where two systems or subjects meet and interact.

#### Multidrug resistance

Resistant to three or more antimicrobial classes.

#### Methicillin-resistant *Staphylococcus aureus*

*Staphylococcus aureus* strains that are resistant to penicillin and β-lactams such as methicillin or oxacillin.

#### Community-associated MRSA infections

MRSA infections in healthy people who have not been hospitalized.

#### Animal

In this review stand for cattle.

### Study eligibility criteria

#### Inclusion criteria

The inclusion criteria for this analysis specifically consisted of the following: (I) articles that presented a clear estimate of the proportion of *S. aureus* separately in animals and humans and implemented within the same article and study year; (II) observational studies that demonstrated the prevalence of *S. aureus* isolates at the human-animal interface and/or work equipment; (III) the human participants were the following populations who have direct contact with animals: dairy farm workers with frequent exposure to dairy animals, livestock owners and slaughterhouse workers, including butchers; (IV) the animal population also included domestic animals that were directly used in dairy cows and selected animals for slaughter; and (IV) the examination or sampling units had to be derived from workers (e.g., hand swabs and nasal swabs), animals (both slaughter and dairy cattle) and work equipment (including knives, milk tanks, and milking buckets).

#### Exclusion criteria

The following types of studies were not included in the analysis: those involving camels or other species, those with unclear or imprecise estimates of bacterial species across the affected host, review articles, Hospital workers, hospital working equipment, duplicates, summary-only studies, qualitative studies, or knowledge, attitude, and practice (KAP) questionnaire-based studies. Book chapters, case reports, editorials, short communications, opinions, or studies without original data. For studies that only used samples from dairies or slaughterhouses, the sample was excluded.

### Data extraction

Data extraction was performed independently by four authors (M.D. and K.G.). The following information was collected from the included studies: the name of the first author and year of publication, study period, study design, study regions, sample source, type of sample collected, diagnostic method, overall examination, and positive events (Table 1).

**Table 1:** Overview of the included studies (*n* = 52).

Author	Study period	Region	Setting/sources	Sample taken	Category	D_x_ method	Total	positive	Prevalence
Marami et al. (40)	NA	Oromia	Dairy farm	Udder milk of cow	Animals	Culture, biochemical	135	101	0.748
Marami et al. (40)	NA	Oromia	Dairy farm	Udders’ swabs	Animals	Culture, biochemical	135	98	0.726
Marami et al. (40)	NA	Oromia	Dairy farm	Milkers’ hand’s swabs	Human	Culture, biochemical	30	25	0.833
Marami et al. (40)	NA	Oromia	Dairy farm	Utensils	Equip	Culture, biochemical	144	18	0.125
Tibebu et al. (23),	2020–2021	AA	Dairy farm	Udder milk of cow	Animals	Culture, biochemical	141	36	0.255
Tibebu et al. (23)	2020–2022	AA	Dairy farm	Udders’ swabs	Animals	Culture, biochemical	40	4	0.100
Tibebu et al. (23)	2020–2023	AA	Dairy farm	Milkers’ hand’s swabs	Human	Culture, biochemical	52	10	0.192
Kalayu et al. (41)	2020	Mekelle	Dairy farm	Udder milk of cow	Animals	Culture, biochemical	385	48	0.125
Kalayu et al. (41)	2020	Mekelle	Dairy farm	Swab	Human	Culture, biochemical	71	22	0.310
Mekuria et al. (42)	2010–2011	AA	Dairy farm	Udder milk of cow	Animals	CMT, biochemical, culture	260	42	0.162
Mekuria et al. (42)	2010–2012	AA	Dairy farm	Nasal swab	Human	Biochemical, culture	68	9	0.132
Beyene et al. (43)	2013–2014	AA	Dairy farm	Udder milk of cow	Animals	CMT, biochemical, culture	72	36	0.500
Beyene et al. (43)	2013–2014	AA	Abattoir	Meat	Animals	Biochemical, culture	121	56	0.463
Ayele et. (44)	2014–2015	Oromia	Dairy farm	Udder milk of cow	Animals	CMT, biochemical, culture	27	3	0.111
Ayele. (44)	2014–2015	Oromia	Dairy farm	Milkers’ hand’s swabs	Human	Biochemical, culture	25	8	0.320
Banu et al. (36)	2020–2021	Oromia	Dairy farm	Udder milk of cow	Animals	CMT, biochemical, culture	212	48	0. 227
Banu et al. (36)	2020–2022	Oromia	Dairy farm	Milkers’ hand’s swabs	Human	Biochemical, culture	44	7	0. 165
Banu et al. (36)	2020–2023	Oromia	Dairy farm	Milkers’ buckets	Equip	Biochemical, culture	55	7	0.127
Geletu et al. (37)	2018–2019	AA	Dairy farm	Udder milk of cow	Animals	CMT, biochemical, culture	125	19	0.152
Geletu et al. (37)	2018–2019	AA	Dairy farm	Fecal sample	Animals	Culture	211	35	0.166
Geletu et al. (37)	2018–2019	AA	Dairy farm	Nasal swab	Animals	Biochemical, culture	211	35	0.166
Geletu et al. (37)	2018–2019	AA	Dairy farm	Milkers’ hand swabs	Human	Biochemical, culture	20	2	0.100
Geletu et al. (37)	2018–2019	AA	Dairy farm	Floor swabs	Equip	Biochemical, culture	20	0	0.000
Geletu et al. (37)	2018–2019	AA	Dairy farm	Bulk milk	Animals	Biochemical, culture	20	6	0.300
Gizaw et al. (45)	2020–2021	AA	Dairy farm	Tank milk	Animals	Gram-stain, biochemical, culture	50	14	0.280
Gizaw et al. (45)	2020–2021	AA	Dairy farm	Milker nasal swab	Human	Gram-stain, biochemical, culture	17	4	0.235
Gizaw et al. (45)	2020–2021	AA	Dairy farm	Udder milk of cow	Animals	Gram-stain, biochemical, culture	297	67	0.226
Gizaw et al. (45)	2020–2021	AA	Dairy farm	Bucket swab	Equip	Gram-stain, biochemical, culture	50	10	0.200
Gizaw et al. (45)	2020–2021	AA	Dairy farm	Milker’s hand swab	Human	Gram-stain, biochemical, culture	50	10	0.200
Gizaw et al. (45)	2020–2021	AA	Dairy farm	Tank swab	Equip	Gram-stain, biochemical, culture	50	10	0.200
Gizaw et al. (45)	2020–2021	AA	Abattoir	Hand swab	Human	Gram-stain, biochemical, culture	37	7	0.189
Gizaw et al. (45)	2020–2021	AA	Abattoir	Slaughter line swab	Equip	Gram-stain, biochemical, culture	37	7	0.189
Gizaw et al. (45)	2020–2021	AA	Abattoir	Abattoir knife swab	Equip	Gram-stain, biochemical, culture	37	5	0.135
Gizaw et al. (45)	2020–2021	AA	Abattoir	Carcass/meat swab	Animals	Gram-stain, biochemical, culture	361	38	0.105
Abunna et al. (46)	2014	Oromia	Dairy farm	Udder milk of cow	Animals	Gram-stain, biochemical, culture	42	5	0.119
Abunna et al. (46)	2014	Oromia	Dairy farm	Tank milk	Animals	Gram-stain, biochemical, culture	9	3	0.333
Abunna et al. (46)	2014	Oromia	Dairy farm	Bucket swab	Equip	Gram-stain, biochemical, culture	9	3	0.333
Abunna et al. (46)	2014	Oromia	Dairy farm	Hand swab	Human	Gram-stain, biochemical, culture	7	3	0.429
Abunna et al. (46)	2014	Oromia	Dairy farm	Nasal swab	Human	Gram-stain, biochemical, culture	9	3	0.333
Abunna et al. (46)	2014	Oromia	Abattoir	meat swab	Animals	Gram-stain, biochemical, culture	66	13	0.197
Abunna et al. (46)	2014	Oromia	Abattoir	Knife swab	Equip	Gram-stain, biochemical, culture	7	5	0.714
Abunna et al. (46)	2014	Oromia	Abattoir	Slaughter line swab	Equip	Gram-stain, biochemical, culture	7	3	0.429
Abunna et al. (46)	2014	Oromia	Abattoir	Hand swab	Human	Gram-stain, biochemical, culture	7	1	0.143
Abunna et al. (46)	2014	Oromia	Abattoir	Nasal swab	Human	Gram staining, biochemical, culture	7	0	0.000
Regasa et al. (47)	2017–2018	Oromia	Dairy farm	Udder milk of cow	Animals	Gram stain, biochemical, culture	183	28	0.153
Regasa et al. (47)	2017–2018	Oromia	Dairy farm	Hand swab	Human	Gram-stain, biochemical, culture	24	6	0.250
Regasa et al. (47)	2017–2018	Oromia	Dairy farm	Bucket swab	Equip	Gram-stain, biochemical, culture	30	6	0.200
Regasa et al. (47)	2017–2018	Oromia	Dairy farm	Towel swab	Equip	Gram-stain, biochemical, culture	10	1	0.100
Adugna et al. (48)	2013–2014	AA	Abattoir	meat	Animals	Gram-stain, biochemical, culture	384	36	0.094
Adugna et al. (48)	2013–2014	AA	Abattoir	Butcher	Human	Gram-stain, biochemical, culture	384	76	0.198
Adugna et al. (48)	2013–2014	AA	Bucher shop	Cutting tables	Equip	Gram-stain, biochemical, culture	40	6	0.150
Adugna et al. (48)	2013–2014	AA	Abattoir	Knife swab	Equip	Gram-stain, biochemical, culture	40	9	0.225

AA stands for Addis Ababa; Dx method stands for diagnosis method; California mastitis test; Equip for equipment; NA for not available.

### Study quality assessment

An independent quality assessment was conducted by two researchers (A.S. and K.G) using the Newcastle–Ottawa Quality Assessment Scale (NOS) (Supplementary material S1).

### Data synthesis and statistical analysis

To determine the pooled prevalence estimate for the *S. aureus* worker-animal-working equipment interfaces in the included studies, a meta-analysis with a random effects approach and a 95% confidence level were used to aggregate the studies. This meta-analysis was conducted using R software version 4.1.3 and included the overall effect size, heterogeneity, and weight of each study, as well as subgroup analysis. The Cochran Q test (reported as *p* value) and the inverse variance index (I^2^) were used to assess the degree of heterogeneity. The I^2^ index, estimated according to the explanation of Thompson and Higgins (49), was used to denote low, moderate, and high heterogeneity, with corresponding I^2^ values of 25, 50, and 75%, respectively. The presence of heterogeneity between studies was assessed using a forest plot. The forest plot diagram shows weights (both random and common effects), effect sizes, and 95% confidence intervals for individual studies (CLs). Additionally, subgroup analyses were performed to examine the prevalence of *S. aureus* based on the source of the pathogen (slaughterhouse or dairy farm). Small study effects and publication bias were visualized using funnel plot diagrams and Egger and Begg tests (50). Egger’s regression test was used to test funnel plot symmetry. A regression model is created with the standardized estimate of the effect size (event rate) as the dependent variable and the reciprocal of the standard error (1/SE) as the independent variable. If the intercept deviates significantly from zero, the estimate of the effect is considered biased (51).

## Results

### Search results

**Figure 1:**
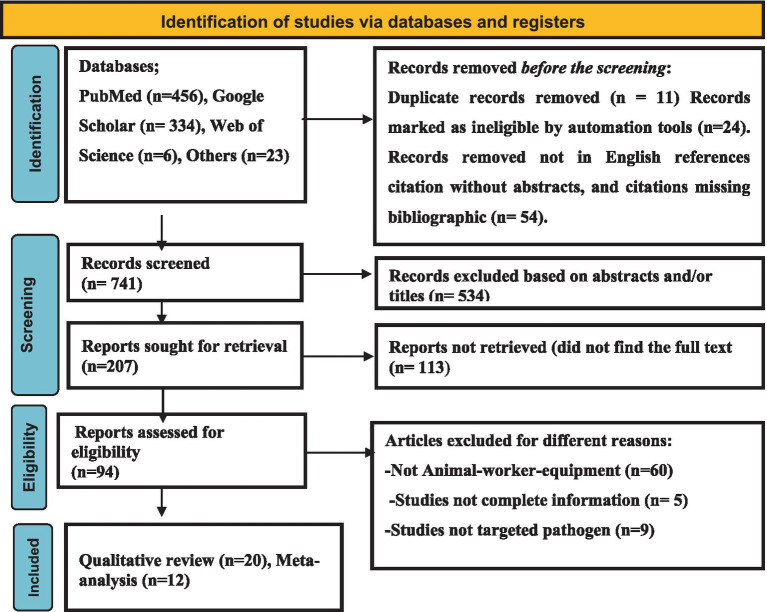
PRISMA flow diagram for investigating the eligible studies.

As shown in Figure 1, a total of 819 articles were browsed through different electronic databases and via other methods. A total of 11 duplicate articles were removed because they were considered ineligible for other reasons (*n* = 78), while 741 records were screened. A total of 534 articles were excluded through title and abstract screening. Two hundred-seven (*n* = 207) articles were retrieved, and 94 were evaluated for eligibility. Finally, 20 qualitative articles and 13 quantitative syntheses were included.

### Characteristics of the included studies

The included studies were conducted in between 2010 and 2023 (Table 1). It should be noted that the same article was utilized multiple times due to different sampling units. The study designs employed were cross-sectional. Of the 13 articles, sex (23, 37, 42, 43, 45, 48) were conducted in Addis Ababa, the capital city of Ethiopia, and five (36, 40, 44, 46, 47) were conducted in the Oromia region. The remaining (41) two (17%) were found in the Tigray region, which is located in the northern part of the country. One of the included studies collected samples from the abattoir (meat), Bucher’s shop (equipment), and the butcher himself (human) (48), while another study collected samples from the environment (floor swabs), workers, and dairy cows (37). Most of the studies focused on collecting specimens from abattoirs, dairy settings, and workers.

### Molecular characterization of isolates

#### Identified genes

Kalayu (41) performed the molecular characterization of isolates of *S. aureus*. A total of 70 isolates were examined for *mecA* and *mecC* possession, 48 of which came from the udder quarters of cows and 22 from the nares of farmers. *MecA* was detected in only one isolate that was taken from a farm worker’s nose. There was no *mecA* recorded from the cows. Furthermore, none of the isolates from humans or cows tested positive for *mecC*. The *mecA*-positive *S. aureus* isolates were identified as *SCCmec type Iva* and *spa type t064* upon further examination. PCR amplification of the seven phenotypically MRSA isolates revealed that 3 (42.9%) of them were found to carry the *femA* gene, and 5 (71.4%) of them were found to carry *mec A* genes, which had molecular weights of 132 bp and 310 bp, respectively.

To further elaborate on the molecular characterization, two articles were included. Seven of the phenotypically MRSA isolates were screened for the presence of the *mecA* and *femA* genes by multiplex PCR according to the procedure described by Johnson et al. (52). The expected amplicon size for *mecA* was 162 bp, while that for *mecC* was 138 bp. in 20 (38.5%) of the isolates. However, only one (5%) of the 20 isolates was found to be positive for the *mecA* gene, and none of the isolates were found to carry the *mecC* gene. The *mecA*-positive isolate was obtained from a raw milk sample (53). Tigabu et al. (23) attempted to detect *mecA*-positive strains from dairy milk but neither found it. However, it should be noted that both studies targeted only *mecA* but not mecC (another gene responsible for methicillin resistance).

### Multidrug-resistant and methicillin-resistant *Staphylococcus aureus*

**Figure 2:**
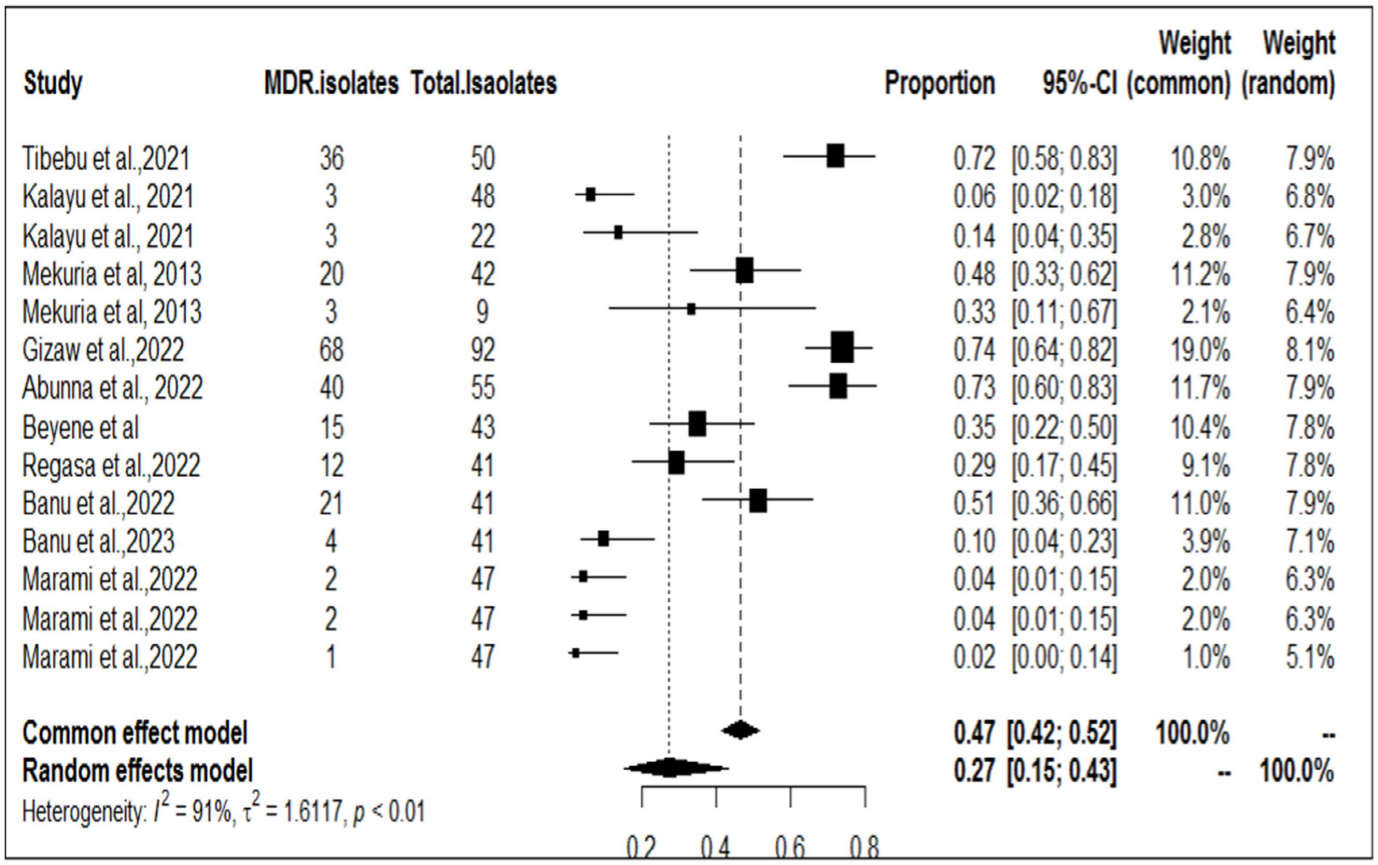
The pooled proportion of *S. aureus* isolates with MDR strains.

Among the studies included in this meta-analysis, nine examined the multidrug resistance profile of *S. aureus*, while five also investigated MERSA (Table 2). Tibebu et al. (23) reported that the highest resistance among the overall isolates was observed against penicillin, with a rate of 47 (94%), followed by ampicillin, which accounted for 46 (92%), and tetracycline, which accounted for 37 (74%). MRSA was detected in 2 (4%) isolates from cow milk samples collected from one dairy farm. Among the nine antibiotic disks used, mono-drug resistance was observed in 1 (2.08%) isolate, while the remaining isolates showed resistance to two, three, four, or five antimicrobials, accounting for 11 (22.92%), 32 (66.67%), 3 (6.25%), or 1 (2.08%) of the samples, respectively. The overall rate of MDR was 72% (ampicillin, penicillin, chloramphenicol, tetracycline, and ciprofloxacin), which was the highest proportion, followed by that reported by Gizaw et al. (56) (68/92, 67%). Among the included studies, Marami et al. (40) reported that five *S. aureus* isolates (two from raw milk, two from utensil swabs, and one from milker’s hand swabs) were resistant to three antimicrobial classes (tetracycline, quinolones, and β-lactams). The highest frequency of MERSA isolates was recorded by Gizaw et al. (45, 56) at (38/92, 41%). To determine the pooled resistance rate of MDR isolates of *S. aureus*, a meta-analysis was conducted across nine studies. The current meta-analysis revealed an overall multidrug resistance rate of 27% (95% CI, 15–43%; Figure 2) in different sampling units.

**Table 2:** Multidrug resistant (MDR) and methicillin-resistant (MR) of *Staphylococcus aureus.*

Author	Sample source	*S. aureus* isolates	No. MRSA	No. MDR Isolates	Antimicrobials rendering MDR
Tibebu et al. (54)	Milk, hand swab	50	2	36	Ampicillin, penicillin, chloramphenicol, tetracycline, ciprofloxacin
Kalayu et al. (41)	Milk sample	48	1	3	Not stated
Kalayu et al. (41)	Nasal swab	22	0	3	Not stated
Mekuria et al. (42)	Dairy farm settings	42	2	20	Tetracycline, quinolones, Penicillin, cephalosporin, cloxacillin, cotrimoxazole
Mekuria et al. (42)	Dairy farm workers	9	4	3	Tetracycline, quinolones, Penicillin, cephalosporin, cloxacillin, cotrimoxazole
Gizaw et al. (45)	Bucket swab, milk	92	38	68	Quinolones, Penicillin, cephalosporin, tetracycline
Abunna et al. (55)	Knife	55	0	40	Ampicillin, cefoxitin, chloramphenicol, cloxacillin, Nalidixic acid, Nitrofurantoin, Streptomycin, penicillin, Tetracycline, Vancomycin
Beyene et al. (43)	Tank milk	43	0	15	Not stated
Regasa et al. (47)	Milk	41	0	12	Not stated
Banu et al. (36)	Milk	41	0	21	Vancomycin, ampicillin, cefotaxime, ceftriaxone, ciprofloxacin, tetracycline, cotrimoxazole
Banu et al. (36)	Human sample	41	0	4	Vancomycin, Ampicillin, cefotaxime, ceftriaxone, ciprofloxacin, tetracycline, cotrimoxazole
Marami et al. (40)	Milk	47	0	2	Tetracycline, quinolones, and β-lactams
Marami et al. (40)	Utensil swab	47	0	2	Tetracycline, quinolones, and β-lactams
Marami et al. (40)	Milker’s hand swab	47	0	1	Tetracycline, quinolones, and β-lactams

MRSA, methicillin-resistant *S. aureus*; MDR, multidrug-resistant.

### Meta-analysis and subgroup analysis

**Figure 3:**
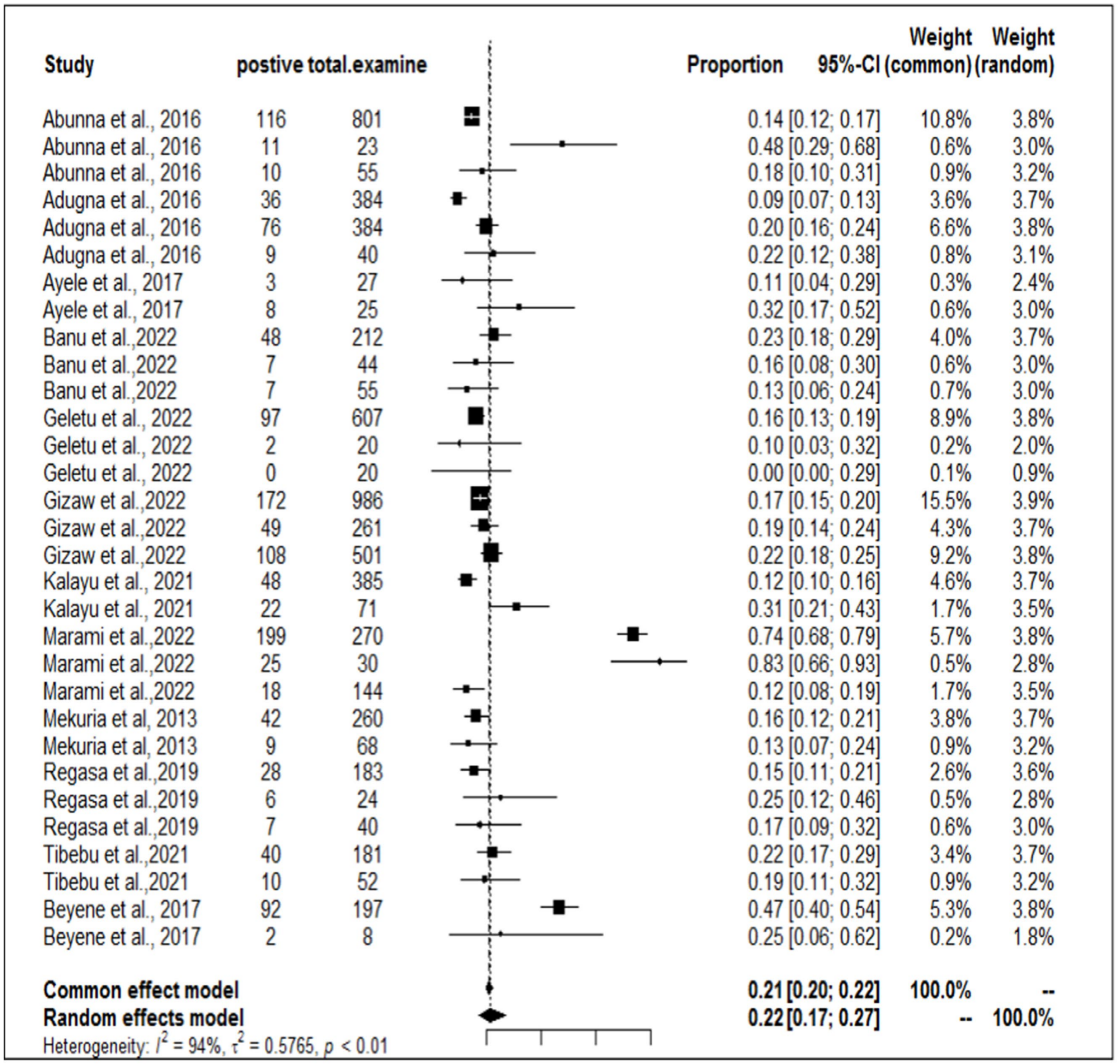
The overall prevalence of *S. aureus* at the worker-animal-equipment interface.

**Figure 4:**
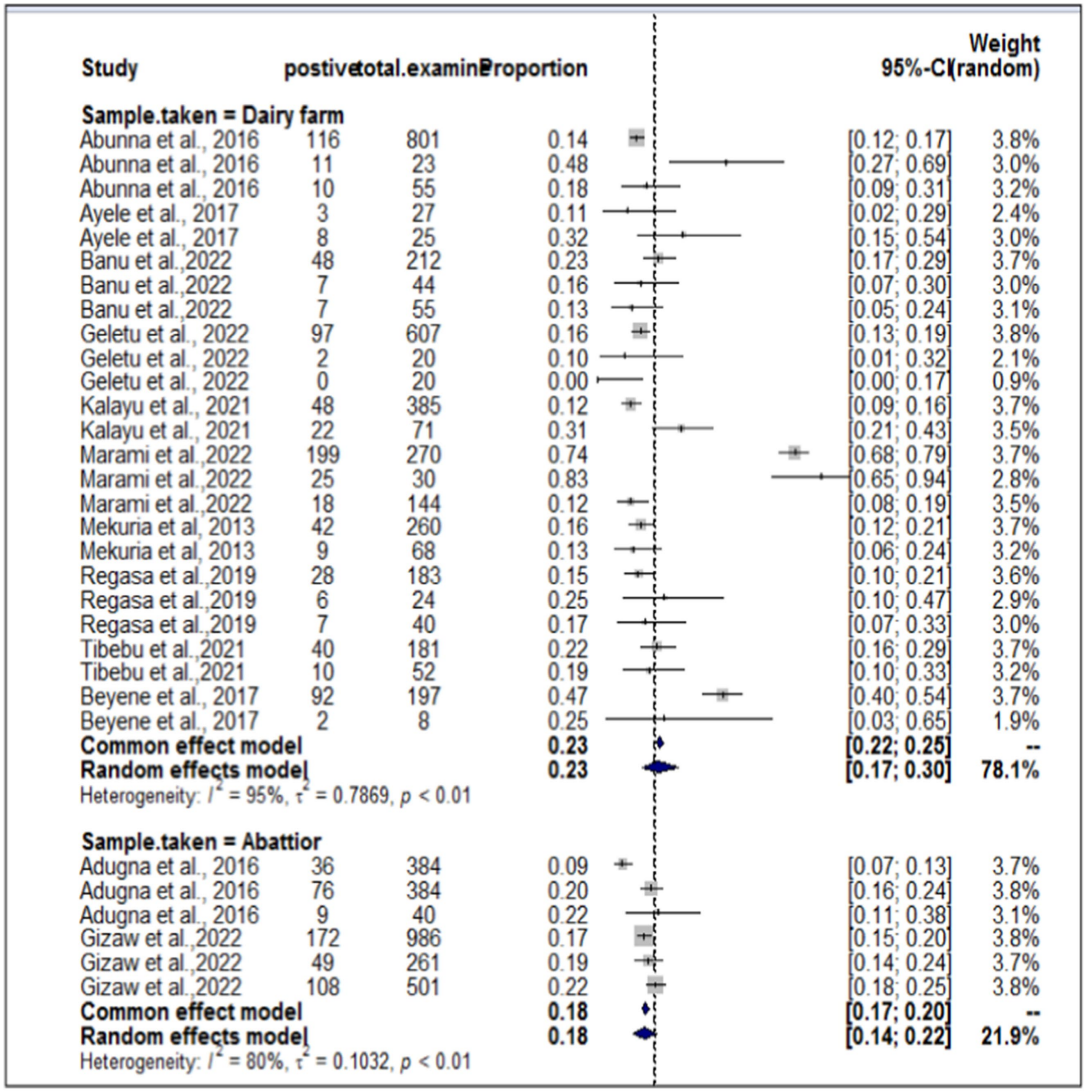
Subgroup analyses by sample source from dairy farms and abattoirs.

In the present meta-analysis, 13 independent articles and 31 dependent articles were included for subgroup analysis. It is important to note that certain articles were utilized multiple times due to their importance in similar years but different sampling units. The included studies demonstrated a high level of heterogeneity (I^2^ = 94%: τ2 = 0.5765; *p* < 0.01), and the estimated pooled prevalence of *S. aureus* at the interface between workers, animals, and working equipment was 22% (95% CI: 17–27%; Figure 3). The variability between studies was statistically significant (Q = 8139.7, DF = 30, *p* < 0.0001). The regression test for funnel plot asymmetry was conducted using a mixed-effects meta-regression model with standard error as the predictor (Eger’s test, b = −1.1073 (CI: −1.6238, −0.5908), z = −0.6382, *p* = 0.5234). Subgroup analyses were performed based on the sources of the samples (abattoir or dairy farm). The subgroup analyses of exploratory outcomes revealed greater heterogeneity across studies on dairy farms (I^2^ = 95%) than on abattoirs (I^2^ = 80%). Similarly, the subgroup analysis indicated that the overall pooled prevalence of *S. aureus* at the interface between animals, workers, and equipment was 23% greater for dairy farm sources (95% CI: 17–30%) than for abattoirs at 18% (95% CI: 14–22%; Figure 4).

**Figure 5:**
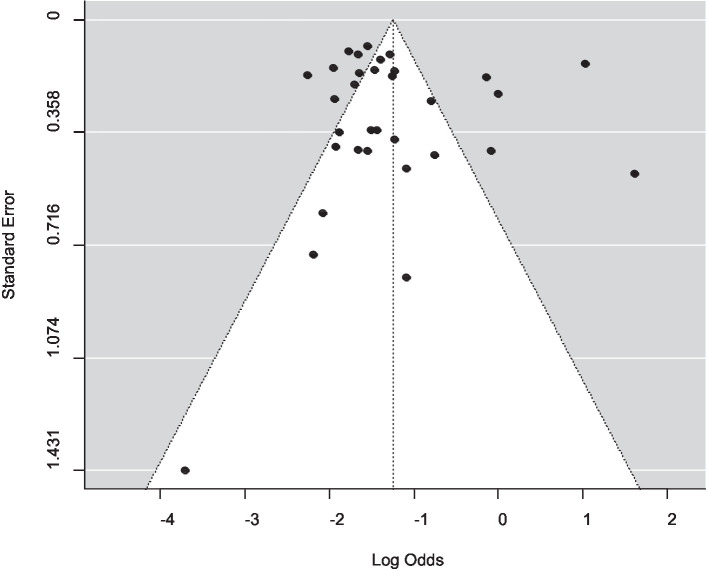
Assessment of publication bias in the included studies.

A funnel plot was used to assess publication bias, which was then supported by the Egger regression test. There was no indication of a symmetrical distribution of articles according to the funnel plot analysis (Figure 5). An analysis of funnel plot asymmetry was performed using a test for small-study effects. The results of Egger’s regression asymmetry, b = −1.1073 (CI: −1.6238, −0.5908; *p* = 0.5234), did not support the existence of publication bias.

### Individual prevalence of *Staphylococcus aureus* in workers, animals, and equipment in the same article

**Figure 6:**
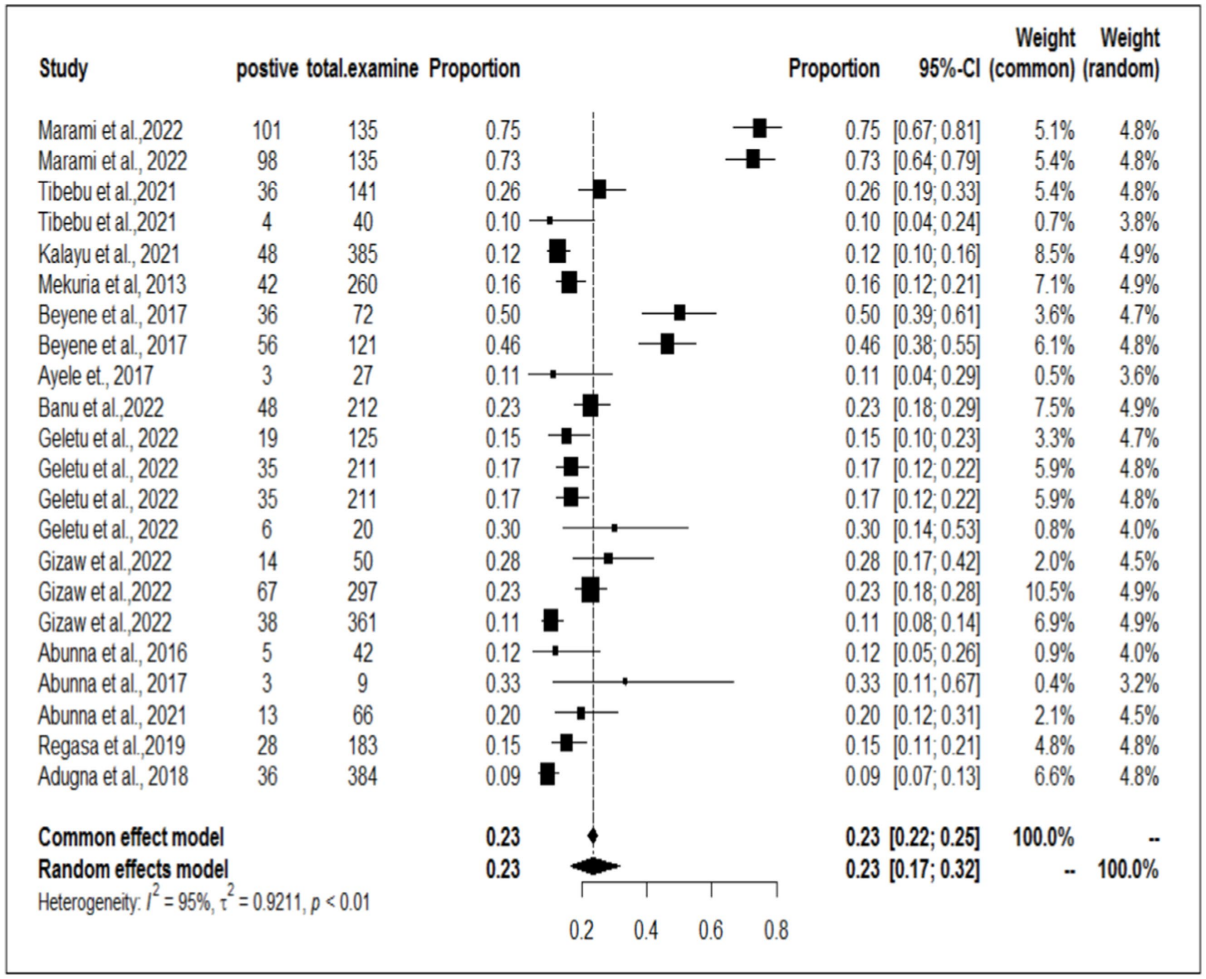
Forest plot of *S. aureus* samples taken from animals.

**Figure 7:**
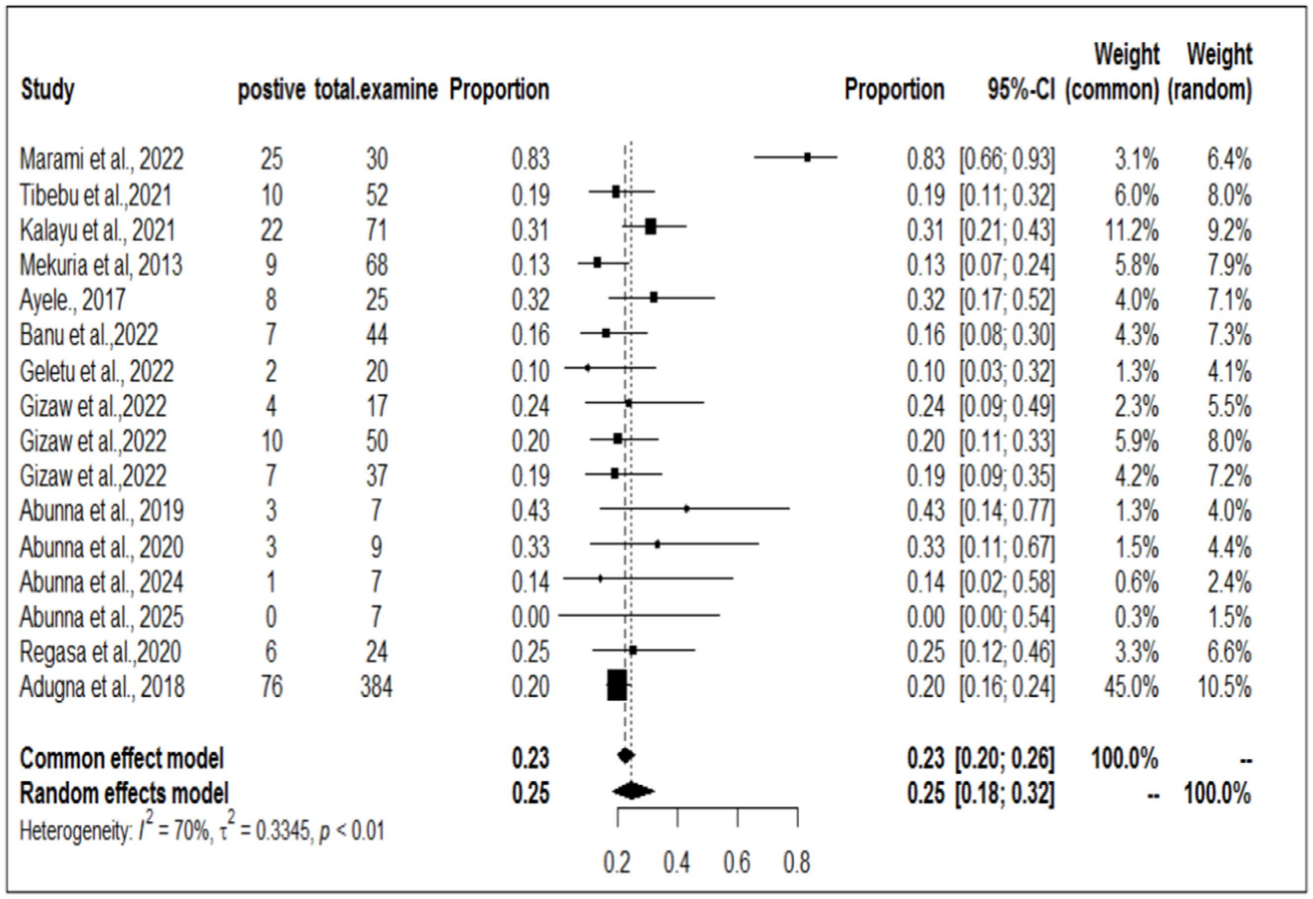
Forest plot of the pooled prevalence of *S. aureus* samples taken from workers.

**Figure 8:**
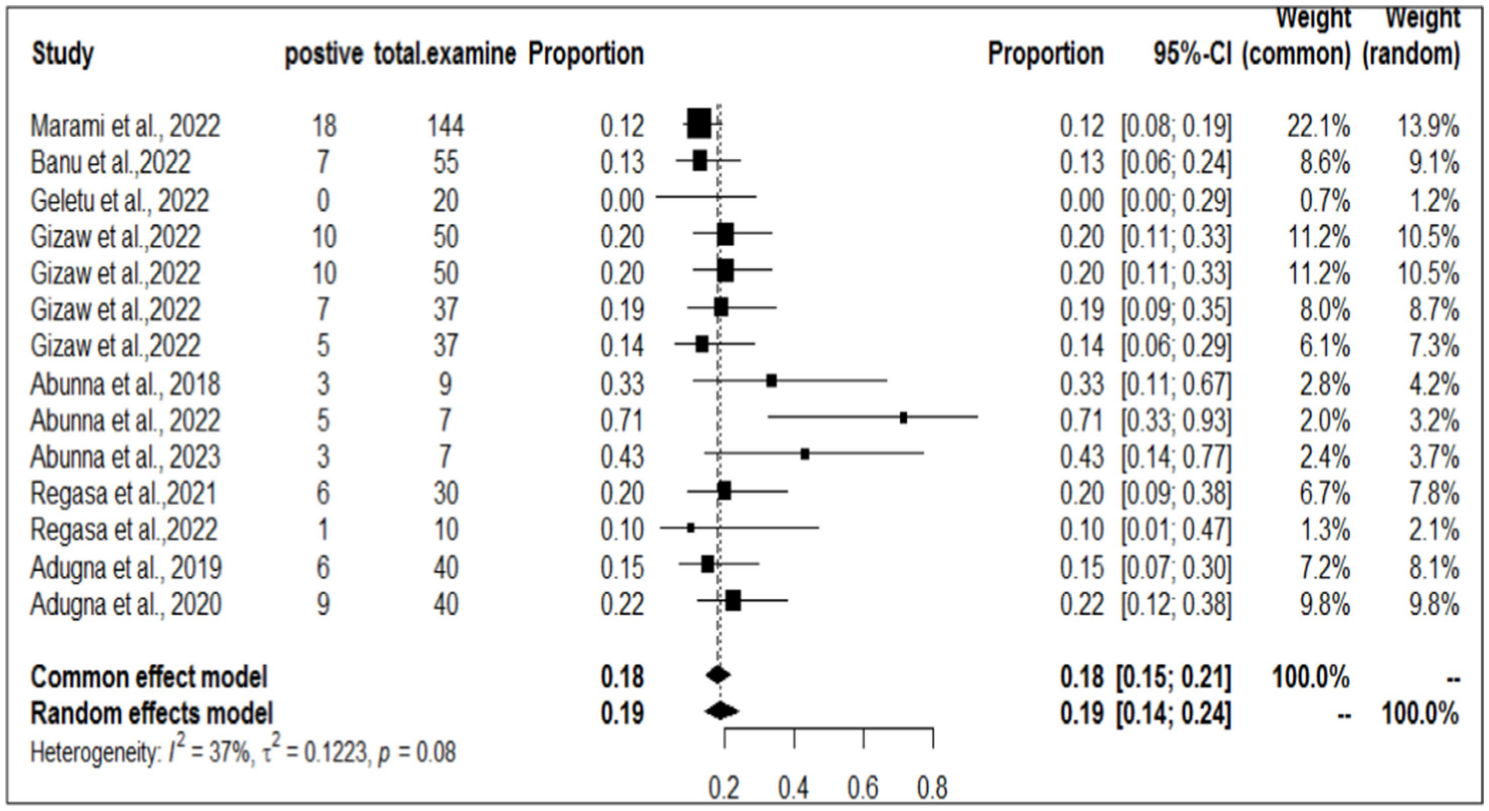
Forest plot of *S. aureus* samples taken from working equipment.

To investigate the prevalence or burden of *S. aureus* in each of the sampling units (animal, workers, and working equipment), a separate analysis of the articles was carried out. Some of the articles were used more than once due to different sample sources. Therefore, based on the separate meta-analysis, the pooled prevalence of *S. aureus* was estimated to be 25% (95% CI; 18–32%) for human/worker samples, 23% (95% CI; 17–32%) for animal sources and 19% (95% CI; 14–24%) for working equipment (Figures 6–8).

### Qualitative assessment of potential factors for *Staphylococcus aureus*

Using logistic regression analysis, Buna’s study (36) demonstrated that milk contamination with *S. aureus* was considerably greater in lactating cows in the older age group, with an odds ratio of 3.91, and implied that lactating cows exhibited a 3.91 times greater risk of being affected by the organism than in the adult and younger age groups. The incidence of *S. aureus* in the milk of cows with numerous parities or calving also differed significantly (*p* = 0.001) and in the late lactation stage (*p* = 0.010). Additionally, there was a strong correlation (*p* < 0.05) between the prevalence of *S. aureus* in milk and hand washing before and after each milking, the frequency of cleaning dairy houses, and the farms’ drainage systems. Furthermore, there was significant variation in the prevalence of bacteria in milk according to age (*p* ≤ 0.001), parity (*p* ≤ 0.001), study area (*p* = 0.035), drainage quality of the milking area (*p* = 0.035), and management system (*p* = 0.035) (54). However, there was no statistically significant correlation (*p* > 0.05) between the prevalence of *S. aureus* and the study towel or udder cleaning interval.

## Discussion

*Staphylococcus* species are prevalent foodborne bacterial pathogens that cause food poisoning in humans when ingested in contaminated foods, including dairy products. The organisms can gain access to raw milk and milk products either by direct excretion from udders with clinical and subclinical staphylococcal mastitis or by contamination from food handlers. The primary aim of interest in this meta-analysis was to estimate the pooled prevalence of *S. aureus* in livestock, livestock-associated workers, and working equipment interfaces in Ethiopia. Our meta-analysis revealed that there was a 22% pooled prevalence of *S. aureus* in the worker-animal and equipment interfaces. The significant increase in the incidence of this pathogen is alarming both for dairy farms and for public health.

The sub-analysis revealed that the pooled prevalence of *S. aureus* at the animal-worker-equipment interface was greater in dairy farm sources, which accounted for 23%, than in abattoir settings, which accounted for 18%. This result contradicted the findings reported by Abunna et al. (55), who reported a greater proportion of *S. aureus* (53.2%) in a slaughterhouse where the percentage of *S. aureus* was slightly greater than that in a dairy farm (44.8%). Another contradictory result was found in the study by Gizaw et al. (56), in which the occurrence of *S. aureus* was significantly greater in meat samples (22.7%) and considerably lower in dairy farms (11.7%) in Ethiopia. According to Tibebu et al. (23), the overall prevalence of *S. aureus* was estimated to be 21.46%. Of these, 25.53% were derived from cow milk, 10% from udder swabs, and 19.23% from hand swabs. On the other hand, Marami et al. (40) reported that 74% of udder milk samples, 72% of udder swabs, and 83% of milker hand swabs were positive for *S. aureus*.

The potential causes for this variation may stem from the inadequate management practices employed by dairy farms and their workers, as well as the insufficient attention given to dairy farms in Ethiopia. These discrepancies could arise from the use of various research methods employed in different studies conducted by multiple researchers. Another possible factor could be attributed to the characteristics of the samples, such as the presence of meat or milk, which may or may not favor the growth and proliferation of *S. aureus*. Moreover, the protocols for processing samples in terms of abattoir settings and dairy farms could introduce significant variations in the proportion of the pathogen in different sampling units. Like other contradictory studies in Ethiopia, the present study depicted a contrary notion in Turkey, as it was reported that meat product samples (48.7%) had a significantly greater prevalence than milk and dairy products (23.2%), implying a widespread distribution of *S. aureus* among diversified food items (57). A converse result might be that the Turkish dairy management system receives great attention throughout the milk and milk product processing chain and provides better treatment options for the pathogen. Moreover, this difference could be attributed to the diverse isolation and identification methods of the agent across different geographical areas as well as to global climatic changes.

The present discovery also indicated that the occurrence of *S. aureus* was greater in human workers (25%) than in animals (23%). The pooled prevalence of *S. aureus* in animals was significantly greater (23%) than that in the study conducted by Marami (40), which revealed that the prevalence of the pathogen in animals was 13.9%. Furthermore, the occurrence of *S. aureus* in animal workers was also found to be greater (25%) than that in Mekuria’s study (42), which reported a 15.5% prevalence of *S. aureus* in milk samples from dairy cows and nasal swabs of farm workers in selected dairy farms around Addis Ababa, Ethiopia. Similarly, our study demonstrated a higher result than the finding documented by Lewis and James (58), where the proportion of *S. aureus* accounted for 17.2% of the cattle, food chain, and human infections in Bishoftu, Ethiopia. However, this meta-analysis result was lower than that of Bendahou et al. (59, 60), who reported that 40% of *S. aureus* isolates were found in dairy products in North Morocco. The lower prevalence of *S. aureus* in the current study compared to the study in northern Morocco could be attributed to the direct collection of milk samples from cows’ udders before contact with milking utensils in this study, which may have reduced the prevalence of *S. aureus* (40).

Risk factors, Tibebu et al. (23) study on *S. aureus* in milk linked the bacteria to various possible factors, including milker age, community awareness of *S. aureus*, antiseptic use before and after milking, barn drainage systems, management systems, prior exposure to mastitis, and the use of drying towels for each udder separately. Only prior mastitis exposure (*p* < 0.05) from these linked factors to the agent had a statistically significant association with the prevalence of *S. aureus*. In Addition, Marami et al. (40) reported that *Staphylococcus* species isolates from dairy cows and smallholders were common in Central Ethiopia. The authors attempted to correlate the disease’s prevalence with several variables, including site town, breed, farm type, cow age, parity, lactation stage, blind test, eating lesion, tick infestation, floor type, and towel usage. Overall, the results of the univariate logistic regression analysis indicated that there was no significant correlation between any of the research variables in cows and the isolation of *Staphylococcus*. Additional research has evaluated the prevalence of this organism in relation to potential risk variables, such as sex, age, farm duty, job experience of the attendees/owners, and parity and lactation status of the cow (41). Carriage of staphylococci in the nasal passages and hands of the milkers are also potential sources of staphylococci in milk.

The prevalence and extent of antimicrobial resistance in veterinary medicine are experiencing a in the global. The spread of staphylococci that are resistant to antimicrobial agents poses a challenge to both human and animal health professionals. It is crucial to monitor and surveil foodborne pathogens and their resistance to antimicrobials in the food supply chain to effectively reduce the risks associated with food-borne hazards. In the present meta-analysis, the collective prevalence of multidrug-resistant strains was 27% in various sampling units on dairy farms and abattoirs. Our findings revealed a significantly lower prevalence than that reported in previous studies conducted in Egypt, with rates of 100, 74, and 80% reported by Seedy et al. (61), Gizaw et al. (56), and Balta et al. (62), respectively. In Korea, Moon et al. (63) reported a prevalence rate of 79%, while Beyene et al.(48)reported a rate of 34.9% in Ethiopia. However, our findings indicated a greater prevalence than that reported in other studies conducted in Ethiopia, such as the 10.42% reported by Tibebu et al. (23) and 16% by Buna et al. (36). This is a result of repeated therapeutic or indiscriminate use of these antibiotics on dairy farms and in humans for the treatment of infections.

The high prevalence of multidrug resistance of *S. aureus* can be attributed to the frequent use of antimicrobials, particularly microorganisms that produce β-lactamase, which inactivates penicillin and related antibiotics. The use of disinfectants containing heavy metals in washing milking equipment could potentially induce antimicrobial resistance in bacteria. It is possible to increase antimicrobial resistance via various ecological settings, by administering subtherapeutic doses, subdose antibiotics in animal feed and water, administering them at the wrong time, and administering them using the wrong route (which impacts bioavailability) (64).

The introduction and propagation of low-grade medications within a nation can also present significant obstacles (65). Additionally, the presence of antimicrobial residues in milk can contribute to the development of antimicrobial resistance in pathogens, which can then be transmitted to different individuals upon consumption (66). The abundance of multidrug-resistant Staphylococcus sp. have been found in milk from farms, meat from abattoirs, equipment, and humans poses a risk to public health.

To effectively control these pathogens at the national level in Ethiopia, veterinary professionals should provide health-related training to para-professionals, such as livestock keepers, attendants, milkers, hygiene workers, and milk collectors. To achieve optimal health outcomes for humans, animals, and the environment, national action plans should prioritize efforts to reduce antimicrobial resistance from livestock to the environment and the community.

## Conclusion and future perspectives

The present meta-analysis revealed that the pooled prevalence of *S. aureus* at the animal-worker-working equipment interface was 22% in Ethiopia. Overall, 25, 23, and 19% of the pathogens were distributed in humans, animals, and working facilities/utensils, respectively. The current meta-analysis also revealed an overall MDR rate of 27% in different sampling units. And, the highest frequency of MRSA isolates was recorded found in the Dairy farm settings (44%). The sub-pooled results also revealed substandard practices for milk, meat, personnel, and equipment hygiene in dairy farms and abattoirs when handling, storing, and processing foods originating from livestock. The contamination rates of food of animal origin (milk and meat), personnel, and working equipment by *S. aureus* are approximately equal, indicating that this particular pathogen is present and circulating at the interface where humans, animals, and working utensils interact. Additionally, it can serve as a useful marker for inadequate hygiene practices and compromised animal food safety. In particular, the frequent cleaning of abattoir workers’ and milkers’ hands could be a contributing factor to the relatively low prevalence of *S. aureus* in abattoirs. The higher pooled prevalence of *S. aureus* in dairy farms, abattoir settings, and workers in this meta-analysis indicates its serious economic, animal welfare, food safety and public health problems and its association with livestock for its transmission between animals and humans in livestock farm settings. Thus, improving hygienic measures on dairy farms and working equipment as well as within abattoirs must be effective in safeguarding the public from the risk of staphylococcal food poisoning and acquiring MDR isolates. Additionally, future research should consider identifying *Staphylococcus* enterotoxins from milk and meat and genes responsible for AMR in Ethiopia.

## Data Availability

The original contributions presented in the study are included in the article/Supplementary material, further inquiries can be directed to the corresponding author.
