# Supplementary material for: Occurrence, multidrug resistance and potential risk factors for Staphylococcus aureus infection at worker-animal and working equipment interfaces: a systematic review and meta-analysis of the Ethiopian literature
Source: Front Public Health. 2024 Aug 16;12:1403012. doi: 10.3389/fpubh.2024.1403012 (PMC11363263; doi:10.3389/fpubh.2024.1403012)
Supplement: Supplementary file 1 [file Table_1.docx]

**Supplementary Table 1.** Quality assessment using the Newcastle–Ottawa scale (NOS) modified for cross-sectional studies for the prevalence of *S. auerus* in the animal-human-working equipment interface..

| S.No. | First author | selection | comparability | outcome | Sum of score |
| --- | --- | --- | --- | --- | --- |
| 1. | Marami et al.,2022 | **** | * | ** | 7 |
| 2. | Tibebu et al.,2021. | *** | ** | *** | 7 |
| 3. | Kalayu et al., 2021] | *** | ** | *** | 8 |
| 4. | Mekuria et al, 2013] | *** | ** | ** | 7 |
| 5. | Beyene et al., 2017 | **** | ** | ** | 8 |
| 6. | Ayele et., 2017] | *** | * | ** | 6 |
| 7. | Banu et al.,2022 | *** | * | *** | 7 |
| 8. | Geletu et al., 2022 | ** | ** | *** | 7 |
| 9. | Gizaw et al.,2022 | *** | * | *** | 7 |
| 10. | Abunna et al., 2016 | **** | ** | ** | 8 |
| 11. | Regasa et al.,2019 | *** | ** | ** | 7 |
| 12. | Adugna et al., 2018. | ** | ** | ** | 6 |

Selection (maximum= 5 star); Comparability (maximum= 2 stars); outcome (maximum= 3 stars)

*Indicates one criteria was fulfill, ** two criteria were fulfill, ***three criteria were ffulfill, and ****four criteria were fulfill
